# The Multi-Targeting Ligand ST-2223 with Histamine H_3_ Receptor and Dopamine D_2_/D_3_ Receptor Antagonist Properties Mitigates Autism-Like Repetitive Behaviors and Brain Oxidative Stress in Mice

**DOI:** 10.3390/ijms22041947

**Published:** 2021-02-16

**Authors:** Nermin Eissa, Karthikkumar Venkatachalam, Petrilla Jayaprakash, Markus Falkenstein, Mariam Dubiel, Annika Frank, David Reiner-Link, Holger Stark, Bassem Sadek

**Affiliations:** 1 Department of Pharmacology & Therapeutics, College of Medicine and Health Sciences, United Arab Emirates University, Al Ain P.O. Box 17666, United Arab Emirates; 201690014@uaeu.ac.ae (N.E.); karthikkumar@uaeu.ac.ae (K.V.); petrilla.jp@uaeu.ac.ae (P.J.); 2 Zayed Center for Health Sciences, United Arab Emirates University, Al Ain P.O. Box 17666, United Arab Emirates; 3 Department of Applied Sciences, College of Arts and Sciences, Abu Dhabi University, Al Ain P.O. Box 59911, United Arab Emirates; 4 Institute of Pharmaceutical and Medicinal Chemistry, Heinrich Heine University Düsseldorf, Universitaetsstr.1, 40225 Düsseldorf, Germany; mafal104@hhu.de (M.F.); mariam.dubiel@hhu.de (M.D.); a.frank@hhu.de (A.F.); David.Reiner@uni-duesseldorf.de (D.R.-L.); stark@hhu.de (H.S.)

**Keywords:** autistic spectrum disorder, BTBR mice, histamine H_3_ receptor antagonist, dopamine D_2_/D_3_R antagonist, repetitive and restricted behavior, anxiety, oxidative stress

## Abstract

Autism spectrum disorder (ASD) is a complex heterogeneous neurodevelopmental disorder characterized by social and communicative impairments, as well as repetitive and restricted behaviors (RRBs). With the limited effectiveness of current pharmacotherapies in treating repetitive behaviors, the present study determined the effects of acute systemic treatment of the novel multi-targeting ligand ST-2223, with incorporated histamine H_3_ receptor (H_3_R) and dopamine D_2_/D_3_ receptor affinity properties, on ASD-related RRBs in a male Black and Tan BRachyury (BTBR) mouse model of ASD. ST-2223 (2.5, 5, and 10 mg/kg, i.p.) significantly mitigated the increase in marble burying and self-grooming, and improved reduced spontaneous alternation in BTBR mice (all *p* < 0.05). Similarly, reference drugs memantine (MEM, 5 mg/kg, i.p.) and aripiprazole (ARP, 1 mg/kg, i.p.), reversed abnormally high levels of several RRBs in BTBR (*p* < 0.05). Moreover, ST-2223 palliated the disturbed anxiety levels observed in an open field test (all *p* < 0.05), but did not restore the hyperactivity parameters, whereas MEM failed to restore mouse anxiety and hyperactivity. In addition, ST-2223 (5 mg/kg, i.p.) mitigated oxidative stress status by decreasing the elevated levels of malondialdehyde (MDA), and increasing the levels of decreased glutathione (GSH), superoxide dismutase (SOD), and catalase (CAT) in different brain parts of treated BTBR mice (all *p* < 0.05). These preliminary in vivo findings demonstrate the ameliorative effects of ST-2223 on RRBs in a mouse model of ASD, suggesting its pharmacological prospective to rescue core ASD-related behaviors. Further confirmatory investigations on its effects on various brain neurotransmitters, e.g., dopamine and histamine, in different brain regions are still warranted to corroborate and expand these initial data.

## 1. Introduction

**Figure 1:**
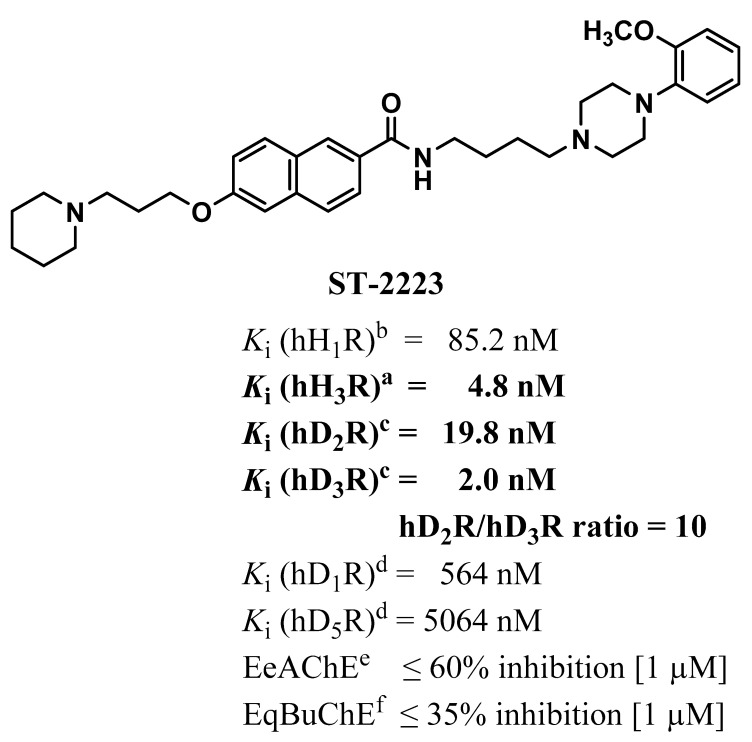
Chemical structure of the multi-targeting ligand ST-2223 with affinities at *h*H_3_R, *h*D_2_R and *h*D_3_R. ^a^ [^3^H]*N*^α^-methylhistamine binding assay, performed with cell membrane preparation of Human embryonic kidney (HEK) cells stably expressing the human H_3_R (*n* = 4) [44,45]. ^b^ [^3^H]pyrilamine binding assay, performed with cell membrane preparation of Chinese hamster ovary (CHO) cells stably expressing the *h*H_1_R (*n* = 2). ^c,d^ Displacement assay was carried out as described previously, using membrane suspension of cell lines stably expressing the human dopamine *h*D_1_Rs and *h*D_5_Rs (HEK) against [^3^H]SCH23390 and *h*D_2_SRs, *h*D_3_Rs (CHO) using [^3^H]spiperone (*n* = 3) [44,45]. ^e^ AChE: Acetylcholine esterase; Ee; electric eel (preliminary data); ^f^ BuChE: Butyrylcholinesterase;Eq: equine (preliminary data) (*n* = 2) [46].

Autism spectrum disorder (ASD) is a highly heterogeneous neurodevelopmental disorder characterized by persistent social interaction deficits coupled with restricted, repetitive patterns of behaviors (RRBs), that are typically diagnosed during early developmental stages [1]. Behavioral observation is the primary diagnostic criteria for these core features of ASD [2,3]. ASD individuals frequently display intense RRBs and thoughts that are reported to be a disturbing aspect for patients of ASD, while also being challenging for their families, influencing their daily functioning [4]. RRBs refer to a broad class of responses characterized by their repetition and rigidity, including motor stereotypies, compulsions, insistence on sameness, circumscribed interests and cognitive inflexibility [5,6,7,8]. Despite its clinical significance and the fact that a great deal of research has been pursued, it has proven difficult to understand the underlying mechanisms contributing to these behaviors, hence RRBs in ASD are still not remediable [9,10]. To date, the FDA has only approved risperidone and aripiprazole, for the management of irritability associated with ASD, but no medications have been approved to treat RRBs in ASD [11]. Hence, understanding the implicated neurochemical mechanisms in brain circuitry that contribute to RRBs, will be critical in developing potential pharmacotherapies [12]. Findings from several studies suggest that RRBs also appear in other CNS disorders, including obsessive-compulsive disorder, Tourette syndrome (TS), and schizophrenia (SCH), all of which are comorbid with ASD [13,14,15,16,17,18]. This overlap suggests that repetitive behavior likely arises from multiple etiologies or sources of CNS disruption. In addition to genetic and environmental factors, growing evidence suggests that a variety of neurotransmitters that have significant roles in initial brain development, such as acetylcholine (ACh), serotonin (5-HT), dopamine (DA), γ-aminobutyric acid (GABA), glutamate (Glu), and histamine (HA) are implicated in the onset and progression of ASD. Thus, substantiating these neurotransmitter systems as being a significant area in studying the etiology of RRBs in ASD [19]. Brain DA and HA have influences on behavior in brain disorders including Alzheimer’s disease, SCH, anxiety, and narcolepsy, all of which show overlap with ASD [20,21,22]. The emergence of the widespread clinical use of antipsychotics that mainly target D_2_ receptors (D_2_Rs), along with the pathophysiological role of dopaminergic system (DS) deficits in ASD, implicates the fundamental role of DA brain functioning [23,24]. Dysregulation of the DS has been reported to alter striatal dopaminergic neurotransmission and DA-dependent behaviors that are implicated in various neuropsychiatric disorders and recently in ASD [22]. ASD is strongly associated with a mutation in the dopamine transporter (DAT) gene *SLC6A3*, which codes for a protein that contributes to regulation of DA levels in the brain [25]. DAT knockout mice (hyperdopaminergic mutant mice) showed greater invariance in complex fixed action patterns, suggesting an association between abnormal DA levels and repetitive behaviors [26]. Furthermore, the siRNA-mediated inhibition of D_2_Rs in the dorsal striatum has been shown to replicate ASD-like phenotypes in D_2_R KO mice [27]. The brain histaminergic system (HS) has also been found to play a critical role in cognition, sleep and several neuropsychiatric disorders, including SCH and TS; comorbidities related to ASD [28]. TS, which is a condition characterized by stereotypies and tics, has been reported to be among the most prevalent comorbid neurodevelopmental disorders with ASD [29], sharing genetic risk factors [30,31]. In recent studies, it was reported that histaminergic signalling abnormalities may contribute to rare diseases such as TS. Moreover, TS has been associated with a premature termination codon (W317X) in the L-histidine decarboxylase (HDC) gene, hence, implicating the HS in the outcome of this syndrome and ASD [32,33,34,35,36,37]. Alteration of the brain’s HA modulatory system has been identified as being a rare genetic cause of tic disorders; indeed, histidine decarboxylase knockout (*Hdc-*KO) mice have been generated, representing a promising model of this pathophysiology [38,39], implying that diminished histaminergic neurotransmission may be related to the exhibited repetitive and tic-like stereotypies [39]. Moreover, alteration in gene expression was found for the histamine-*N*-methyltransferase enzyme (HNMT, an enzyme responsible for the metabolism of central HA) and for histamine receptor (HR) subtypes (H_1_-H_3_R) in ASD [28]. Based on these preclinical experimental outcomes, the central H_3_Rs—as auto- and hetero-receptors that regulate the biosynthesis and release of HA and several neurotransmitters, including DA—are suggested to be attractive targets for developing novel H_3_R antagonists/inverse agonists, that may be potential therapeutics for the treatment of several neurological multi-neurotransmitter disorders, including cognitive impairments and RRBs. Additionally, the implication of a variety of neurotransmitter systems, such as ACh, 5-HT, DA, GABA, Glu, and HA, in the onset and progression of ASD, has suggested that the recent advances in developing novel agents with multiple pharmacological effects as being a promising strategy for the treatment of multifactorial diseases, such as ASD [19]. Therefore, the present study aimed to investigate the effects of the newly developed multiple-targeting ligand ST-2223 [*N*-(4-(4-(2-methoxyphenyl)piperazin-1-yl)butyl)-6-(3-(piperidin-1-yl)propoxy)-2-naphthamide] with high in vitro H_3_R affinity (*h*H_3_R *K*_i_ = 4.8 nM) and potent *h*D_2_/*h*D_3_R affinity (*h*D_2_R *K*_i_ = 19.8 nM, *h*D_3_R *K*_i_ = 2.0 nM), with a preference for *h*D_3_R (ratio *K*i values *h*D_2_R/*h*D_3_R:10) (Figure 1) on RRBs in the Black and Tan BRachyury (BTBR) mouse model of ASD. ST-2223 combines the classical non-imidazole H_3_R pharmacophore in an overlapping integration with the D_3_R/D_2_R ligand BP 897 [40,41,42,43]. Although a small reduction in affinities to DR subtypes is observed, this novel compound maintains affinities at the desired targets in a low nanomolar concentration range.

BTBR is an idiopathic model of ASD, that naturally displays deficits in social interactions, in addition to RRBs including cognitive inflexibility, elevated grooming behavior and marble burying, when compared to B6 mice [47,48,49]. To understand whether ST-2223 treatment has a more general effect on motor behavior, the effect of ST-2223 on locomotor activity was also measured in both mice strains in order to simultaneously rule out possible intrinsic impairment of spontaneous locomotor activity that might mask anxiety parameters [50]. Moreover, since oxidative stress has been shown to be closely related with ASD [51,52], further assessment of the neuroprotective effects of ST-2223 on ASD-like RRBs in BTBR mice compared to B6 mice was carried out. This was due to the fact that BTBR mice have been reported to have elevated levels of oxidative stress with a deficient enzymatic anti-oxidant response that is suggested to be associated with the exaggerated repetitive behavior. To comprehend our observations, the ability of CNS-penetrant H_3_R agonist (*R*)-α-methylhistamine (RAM) to counteract the ST-2223-provided H_3_R antagonist effects was assessed in order to elucidate the possible involvement of brain HA and DA in the enhancements observed by ST-2223.

## 2. Results

### 2.1. Marble Burying Behavior 

**Figure 2:**
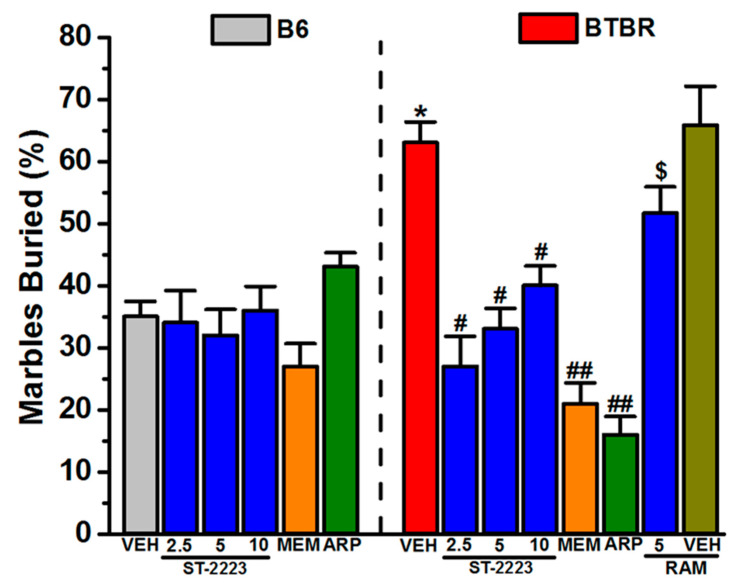
ST-2223 treatment attenuates marble burying in Black and Tan BRachyury (BTBR) mice. Percentage of marbles buried was assessed in B6 and BTBR mice. Each mouse received an i.p. injection of vehicle, ST-2223 (2.5, 5.0, or 10 mg/kg, i.p.), memantine (MEM, 5 mg/kg, i.p.), or aripiprazole (ARP, 1 mg/kg, i.p.) 30–45 min prior to marble exposure. BTBR mice buried significantly more marbles compared to B6 mice. Vehicle or ST-2223 treatment did not significantly affect marble burying in B6 mice. ST-2223 (2.5, 5, and 10 mg/kg, i.p.) and MEM significantly decreased marble burying in BTBR mice. The effects of systemic co-injection of (*R*)-α-methylhistamine (RAM,(10 mg/kg, i.p.) on the ST-2223 (5 mg)-induced decrease in stereotyped repetitive and compulsive behaviors of BTBR mice were also assessed. Data are expressed as the mean ± SEM (*n* = 5). * *p* < 0.05 vs. vehicle-treated B6 mice. ^#^ *p* < 0.05 vs. vehicle-treated BTBR mice. ^##^ *p* < 0.01 vs. vehicle-treated BTBR mice. ^$^ *p* < 0.05 vs. ST-2223 (5 mg)-treated BTBR mice.

The effects of systemic injection of ST-2223 (2.5, 5, and 10 mg/kg, i.p.), memantine (MEM) (5 mg/kg, i.p.) and aripiprazole (ARP) (1 mg/kg, i.p.) on marble burying behavior in BTBR and B6 mice are shown in Figure 2. The results of a two-way ANOVA showed that there was a significant main effect for strain (*F*_(1,48)_ = 3.08, *p* < 0.01), treatment (*F*_(5,48)_ = 4.52, *p* < 0.01) and also for the strain × treatment interaction (*F*_(5,48)_ = 3.16, *p* < 0.05) (Figure 2). Post hoc tests revealed that vehicle-treated BTBR (63.00 ± 3.34 %) buried significantly more marbles compared to B6 mice (35.00 ± 2.45 %) with values of (*F*_(1,8)_ = 7.36, *p* < 0.05). ST-2223 (2.5, 5, and 10 mg/kg, i.p.) significantly decreased the percentage of marbles buried in BTBR mice with values of (*F*_(1,8)_ = 7.38, *p* < 0.05) (*F*_(1,8)_ = 8.52, *p* < 0.05), and (*F*_(1,8)_ = 7.16, *p* < 0.05), respectively. Moreover, no significant differences in the percentage of marbles buried was observed between the three doses of ST-2223, with values of (*F*_(1,8)_ = 0.84, *p* = 0.39) for 2.5 mg/kg versus 5 mg/kg, (*F*_(1,8)_ = 1.85, *p* = 0.21) for 5 mg/kg versus 10 mg/kg, and (*F*_(1,8)_ = 4.07, *p* = 0.09) for 2.5 mg/kg versus 10 mg/kg (Figure 2). Furthermore, the reference drugs MEM (5 mg/kg, i.p.) and ARP (1 mg/kg, i.p.), significantly reduced the percentage of marbles buried with values of (*F*_(1,8)_ = 39.28, *p* < 0.01) and (*F*_(1,8)_ = 73.63, *p* < 0.01), respectively. In B6 mice, ST-2223 did not alter the percentage of marbles buried (all *p*’s > 0.05). However, there was a trend for MEM to reduce marble burying, although systemic treatment with 5 mg/kg was not significant when compared to that of the vehicle treatment (*p* > 0.05). As depicted in Figure 2 and in the post hoc analyses observations, the ST-2223 (5 mg)-stimulated decrease in the percentage of buried marbles was reversed by co-administration with RAM (10 mg/kg, i.p.), with values of (*F*_(1,8)_ = 5.88; *p* < 0.05), as compared to the ST-2223 (5 mg)-treated BTBR mice (Figure 2). Interestingly, subchronic systemic pretreatment of BTBR mice with RAM (10 mg/kg, i.p.) did not alter the behaviors of tested mice in the marble burying task (MBT), with values of (*F*_(1,8)_ = 0.12; *p* = 0.77), as compared to the saline-treated BTBR mice (Figure 2).

### 2.2. Spontaneous Self-Grooming Behavior

**Figure 3:**
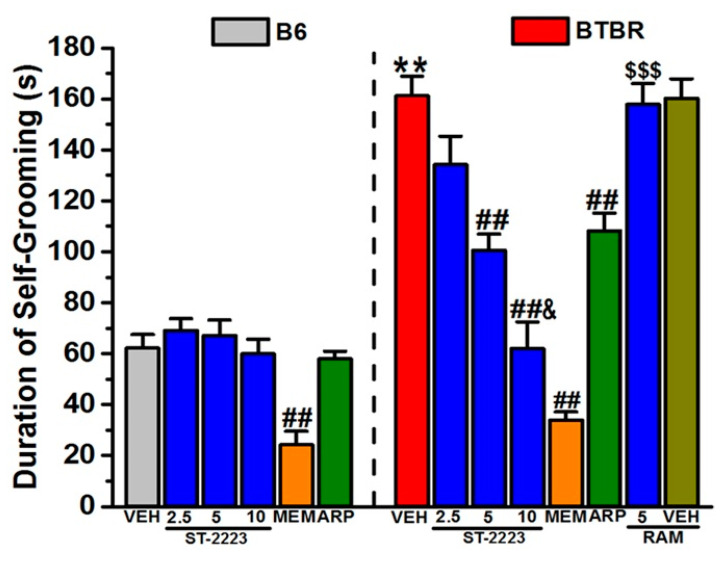
ST-2223 treatment mitigates spontaneous self-grooming behavior in BTBR mice. Self-grooming behavior was measured in B6 and BTBR mice. Each mouse received an i.p. injection of vehicle, ST-2223 (2.5, 5.0, or 10 mg/kg, i.p.), MEM (5 mg/kg, i.p.), or ARP (1 mg/kg, i.p.) 30–45 min prior to self-grooming behavior assessment. BTBR mice spent significantly more time grooming compared to that of B6 mice. Vehicle, ST-2223, MEM, or ARP treatment did not affect spontaneous self-grooming behavior in B6 mice. ST-2223 (5 and 10 mg/kg, i.p.), and MEM (5 mg/kg, i.p.) significantly decreased self-grooming behavior in BTBR mice. ARP (1 mg/kg, i.p.) failed to significantly alter the increased self-grooming behavior of BTBR mice. Effects systemic co-injection of (*R*)-α-methylhistamine (RAM, 10 mg/kg, i.p.) on the ST-2223 (5 mg)-induced decrease in self-grooming behavior of BTBR mice were also assessed. Data are expressed as the mean ± SEM time spent grooming all body regions (*n* = 5). ** *p* < 0.01 vs. vehicle-treated B6 mice. ^##^ *p* < 0.01 vs. vehicle-treated BTBR mice. ^&^ *p* < 0.05 vs. ST-2223 (5 mg)-treated BTBR mice. ^$$$^ *p* < 0.001 vs. ST-2223 (5 mg)-treated BTBR mice.

The effects on spontaneous self-grooming in BTBR and B6 mice are shown in Figure 3. The results of the two-way ANOVA showed that there was a significant main effect for strain, treatment and strain × treatment interaction, with values of (*F*_(1,48)_ = 97.54, *p* < 0.01), (*F*_(5,48)_ = 30.85, *p* < 0.01), and (*F*_(5,48)_ = 11.38, *p* < 0.01), respectively. Post hoc tests revealed that vehicle-treated BTBR (161.20 ± 7.69 s) spent significantly more time grooming when compared to B6 mice (62.20 ± 5.32 s), with values of (*F*_(1,8)_ = 81.69, *p* < 0.01). ST-2223 (5 and 10 mg/kg, i.p.) significantly, and dose-dependently, decreased the time spent self-grooming in BTBR mice, with values of (*F*_(1,8)_ = 29.43, *p* < 0.01) and (*F*_(1,8)_ = 29.56, *p* < 0.01), respectively. However, ST-2223 (2.5 mg/kg, i.p.) failed to significantly mitigate the self-grooming behavior of treated BTBR mice (*p* = 0.23). Interestingly, the reducing effect observed with 10 mg/kg of ST-2223 on self-grooming behavior was significantly higher than that witnessed with the lower dose (5 mg/kg) of the same compound, with values of (*F*_(1,8)_ = 7.97, *p* < 0.05). The reference drugs MEM (5 mg/kg, i.p.) and ARP (1 mg/kg, i.p.) significantly reduced the self-grooming time of BTBR mice with values of (*F*_(1,8)_ = 186.96, *p* < 0.01) and (*F*_(1,8)_ = 20.89, *p* < 0.01), respectively (Figure 3). In B6 mice, ST-2223 (2.5, 5, and 10 mg/kg, i.p.) and ARP (1 mg/kg, i.p.) did not alter the time spent self-grooming (all *p’s* > 0.05). However, MEM (5 mg/kg, i.p.) significantly reduced the self-grooming time of treated B6 mice with values of (*F*_(1,8)_ = 19.50, *p* < 0.01) (Figure 3). As depicted in Figure 3, and following the post hoc analyses of the observed results, the ST-2223 (5 mg)-induced decrease in the duration of time spent displaying self-grooming behaviors, was nullified by co-administration with RAM (10 mg/kg, i.p.), with values of (*F*_(1,8)_ = 27.52; *p* < 0.001), as compared to the ST-2223 (5 mg)-treated BTBR mice (Figure 3). Interestingly, systemic pretreatment of BTBR mice with RAM (10 mg/kg, i.p.) did not alter the behaviors of tested mice in the MBT, with values of (*F*_(1,8)_ = 0.11; *p* = 0.72) as compared to the vehicle-treated BTBR mice (Figure 3).

### 2.3. Spontaneous Alternation Behavior

**Figure 4:**
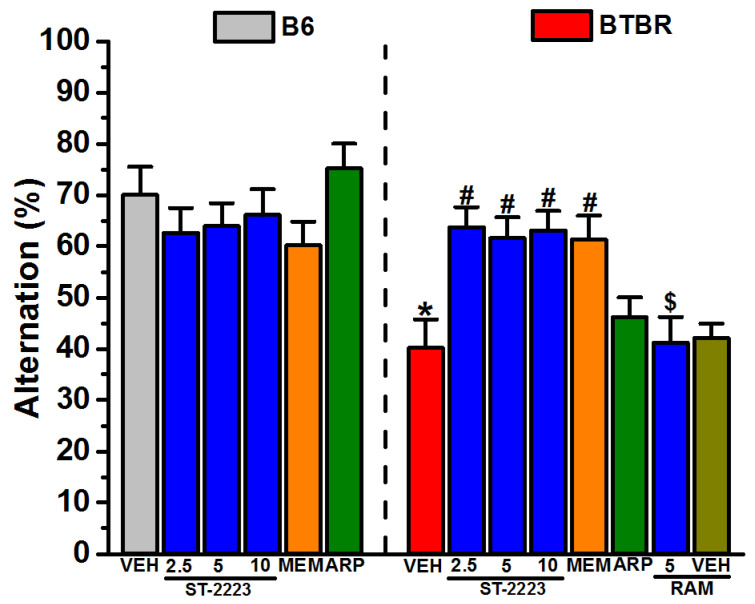
ST-2223 treatment enhances spontaneous alternation behavior in BTBR mice. Spontaneous alternation behavior was measured in B6 and BTBR mice. Each mouse received an i.p. injection of vehicle, ST-2223 (2.5, 5.0, or 10 mg/kg, i.p.), MEM (5 mg/kg, i.p.), or ARP (1 mg/kg, i.p.) 30–45 min prior to assessment of alternation behavior. BTBR mice demonstrated a significantly reduced percentage of spontaneous alternation behavior compared to that of B6 mice. Vehicle, ST-2223, MEM, or ARP treatment did not affect spontaneous alternation behavior in B6 mice. ST-2223 (2.5, 5 and 10 mg/kg, i.p.) and MEM (5 mg/kg, i.p.) significantly mitigated the decreased alteration behavior in BTBR mice. ARP (1 mg/kg, i.p.) failed to alter the decreased alternation behavior of BTBR mice. The effects of systemic co-injection of RAM (10 mg/kg, i.p.) on the ST-2223 (5 mg)-induced enhancement of the alternation behavior of BTBR mice were also assessed. Data are expressed as the mean ± SEM percentage of spontaneous alternation (*n* = 5). * *p* < 0.05 vs. vehicle-treated B6 mice. ^#^ *p* < 0.05 vs. vehicle-treated BTBR mice. ^$^ *p* < 0.05 vs. ST-2223 (5 mg)-treated BTBR mice.

Figure 4 shows the results observed for the spontaneous alternation of BTBR and B6 mice. The results of a two-way ANOVA showed that there was a significant main effect for strain, treatment and strain × treatment interaction, with values of (*F*_(1,48)_ = 15.06, *p* < 0.01), (*F*_(5,48)_ = 16.04, *p* < 0.01), and (*F*_(5,48)_ = 5.22, *p* < 0.01), respectively. Post hoc tests revealed that vehicle-treated BTBR displayed a significantly reduced spontaneous percentage of alternation when compared with vehicle-treated B6 mice, with values of (*F*_(1,8)_ = 14.56, *p* < 0.01). ST-2223 (2.5, 5 and 10 mg/kg, i.p.) and MEM (5 mg/kg, i.p.) significantly increased the percentage of spontaneous alternation in BTBR mice, with values of (*F*_(1,8)_ = 11.23, *p* < 0.05), (*F*_(1,8)_ = 7.20, *p* < 0.05), (*F*_(1,8)_ = 11.29, *p* < 0.05), and (*F*_(1,8)_ = 8.47, *p* < 0.05), respectively (Figure 4). However, ARP (1 mg/kg, i.p.) failed to significantly mitigate the decreased percentage of alternation in BTBR mice (*p* = 0.05) (Figure 4). Moreover, no significant differences in the percentage of spontaneous alternation were observed between the three doses of ST-2223 used, with values of (*F*_(1,8)_ = 0.13, *p* = 0.72) for 2.5 mg/kg versus 5 mg/kg, (*F*_(1,8)_ = 0.07, *p* = 0.80) for 5 mg/kg versus 10 mg/kg, and (*F*_(1,8)_ = 0.01, *p* = 0.92) for 2.5 mg/kg versus 10 mg/kg (Figure 4). In B6 mice, ST-2223 (2.5, 5, and 10 mg/kg, i.p.), MEM (5 mg/kg, i.p.) and ARP (1 mg/kg, i.p.) did not alter the percentage of spontaneous alternation (all *p*’s > 0.05) (Figure 4). As depicted in Figure 4, and following the post hoc analyses of detected results, the ST-2223 (5 mg)-induced enhancement in the alternation of BTBR mice was entirely reversed by co-administration with RAM (10 mg/kg, i.p.), with values of (*F*_(1,8)_ = 8.89; *p* < 0.05) as compared to the ST-2223 (5 mg)-treated BTBR mice (Figure 4). Interestingly, systemic pretreatment of BTBR mice with RAM (10 mg/kg, i.p.) did not alter the behavior of tested mice in the MBT, with values of (*F*_(1,8)_ = 0.09; *p* = 0.78), as compared to the vehicle-treated BTBR mice (Figure 4).

### 2.4. Locomotor Activity and Anxiety Levels

**Figure 5:**
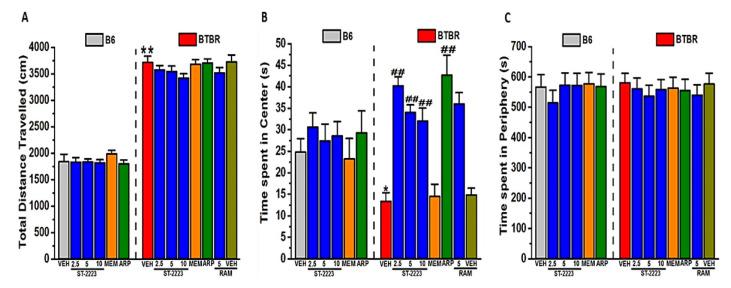
ST-2223 treatment did not affect locomotor activity in BTBR mice. Locomotor activity was assessed in B6 and BTBR mice. Each mouse received an i.p. injection of vehicle, ST-2223 (2.5, 5.0, or 10 mg/kg, i.p.), MEM (5 mg/kg, i.p.), or ARP (1 mg/kg, i.p.) 30–45 min prior to locomotion was measured. BTBR mice travelled significantly greater distances compared to those of B6 mice (**A**). Vehicle, ST-2223, MEM, or ARP treatment did not affect locomotor activity in B6 or BTBR mice (**A**,**C**). BTBR mice spent significantly less time in the center of the arena compared to that of B6 mice (**B**). ST-2223 (2.5, 5 and 10 mg/kg, i.p.) and ARP (1 mg/kg, i.p.) significantly mitigated the decreased amount of time spent by BTBR mice in the center. MEM (5 mg/kg, i.p.) failed to alter the decreased amount of time BTBR mice spent in the center. Data are expressed as the mean ± SEM (*n* = 5). * *p* < 0.05 vs. vehicle-treated B6 mice. ** *p* < 0.01 vs. vehicle-treated B6 mice. ^##^ *p* < 0.01 vs. vehicle-treated BTBR mice.

The findings observed for locomotor activity in B6 and BTBR mice are shown in Figure 5. For the total distance travelled, there was a significant effect of strain (*F*_(1,48)_ = 1059.37, *p* < 0.01), but there was no significant effect for treatment or strain × treatment interaction (*p*’s > 0.05) (Figure 5A). Post hoc tests revealed that vehicle-treated BTBR showed a significant increase in the distance travelled when compared with vehicle-treated B6 mice, with values of (*F*_(1,8)_ = 95.63, *p* < 0.01). B6 and BTBR mice pretreated with ST-2223 (2.5, 5 and 10 mg/kg, i.p.), MEM (5 mg/kg, i.p.), or ARP (1 mg/kg, i.p.) did not show any alteration in the total distance travelled (all *p*’s > 0.05) (Figure 5A). The observed effects of systemic injection of the vehicle, ST-2223 (2.5, 5, or 10 mg/kg, i.p.), MEM or ARP on the time B6 and BTBR mice spent in the center (Figure 5B) and in the periphery (Figure 5C) in the open field test (OFT) are shown. For the time spent in the center, there was a no significant effect of strain (*p* > 0.05), but there was significant effect for treatment and the strain × treatment interaction, with values of (*F*_(5,48)_ = 8.01, *p* < 0.01), and (*F*_(5,48)_ = 3.56, *p* < 0.01), respectively (Figure 5B). Post hoc tests revealed that vehicle-treated BTBR spent significantly less time in the center of the arena when compared with vehicle-treated B6 mice, with values of (*F*_(1,8)_ = 9.11, *p* < 0.05). ST-2223 (2.5, 5 and 10 mg/kg, i.p.) and ARP (1 mg/kg, i.p.) significantly increased the amount of time BTBR mice spent in center, with values of (*F*_(1,8)_ = 33.36, *p* < 0.01), (*F*_(1,8)_ = 46.76, *p* < 0.01), (*F*_(1,8)_ = 21.29, *p* < 0.01), and (*F*_(1,8)_ = 12.11, *p* < 0.01), respectively (Figure 5B). However, MEM (5 mg/kg, i.p.) failed to significantly mitigate the reduced time spent in the center in BTBR mice (*p* = 0.78) (Figure 5B). In B6 mice, ST-2223 (2.5, 5, and 10 mg/kg, i.p.), MEM (5 mg/kg, i.p.) and ARP (1 mg/kg, i.p.) did not alter the amount of time spent in the central arena (all *p*’s > 0.05) (Figure 5B). The observed effects of systemic administration of the vehicle, ST-2223 (2.5, 5, or 10 mg/kg, i.p.), MEM or ARP on the amount of time B6 and BTBR mice spent in the periphery—tested in the OFT—are shown in Figure 5C. For the time spent in the periphery, there was no significant effect for strain, nor for treatment or strain × treatment interaction (all *p*’s > 0.05) (Figure 5C). Notably, post hoc analyses of the detected results revealed that the ST-2223 (5 mg)-induced increase in time spent in the center of the arena was not reversed by co-administration with RAM (10 mg/kg, i.p.), with values of (*F*_(1,8)_ = 0.43; *p* = 0.53), as compared to the ST-2223 (5 mg)-treated BTBR mice (Figure 5B). Interestingly, systemic pretreatment of BTBR mice with RAM (10 mg/kg, i.p.) alone, did not induce any alternation in the amount of time tested BTBR mice spent in the center, with values of (*F*_(1,8)_ = 0.27; *p* = 0.62), as compared to the saline-treated BTBR mice (Figure 5B).

### 2.5. Levels of Oxidative Stress Markers in Different Brain Parts of Treated BTBR Mice

Statistical analysis showed that MDA was significantly increased in the four assessed brain parts (all *p* values < 0.05), and GSH, SOD and CAT were significantly reduced in these brain parts (all *p* values < 0.05) of BTBR mice compared to B6 mice (Table 1). However, the cerebellum of BTBR mice that were pretreated with ST-2223 (5 mg/kg, i.p.) displayed a significant reduction in MDA in all regions assessed (all *p* values < 0.05), and this ST-2223-induced modulation of MDA was reversed when RAM (10 mg/kg, i.p.) was co-administered (all *p* values < 0.05). Moreover, the observed results showed that systemic pretreatment with ST-2223 significantly increased the decreased levels of GSH in the cerebellum, prefrontal cortex and striatum (all *p* values < 0.05), without appreciable effects on MDA levels in the hippocampus. Interestingly, the enhancing effects observed for MDA were counteracted following co-administration with RAM but only in the prefrontal cortex and striatum (all *p* values < 0.05) (Table 1). Moreover, systemic administration of ST-2223 (5 mg/kg, i.p.) significantly increased the decreased level of SOD in the cerebellum (*p* < 0.05), without any appreciable effects on SOD in the hippocampus, prefrontal cortex or striatum. However, ST-2223 significantly increased the decreased levels of CAT in hippocampus, prefrontal cortex and striatum (all *p* values < 0.05), without any appreciable effects on CAT in the cerebellum. Similarly, the cerebellum of BTBR mice that were pretreated with ARP (1 mg/kg, i.p.) or MEM (5 mg/kg, i.p.) showed a significant reduction in MDA in all four brain parts (all *p* values < 0.05), a significant increase in GSH in the cerebellum, hippocampus and striatum, a significant increase in the cerebellum, prefrontal cortex and striatum, and a significant increase in CAT (only for ARP) in the cerebellum and striatum, without any appreciable effects for MEM on the assessed levels of CAT in all four brain regions (Table 1).

## 3. Discussion

### 3.1. In Vitro Affinities for hH_1_Rs, hH_3_Rs, hD_1_Rs, hD_2_Rs, hD_3_Rs and hD_5_Rs 

The novel multiple-targeting test compound ST-2223 was evaluated for its in vitro H_3_R affinity by [^3^H]*N*^α^-methylhistamine displacement assays on membrane preparations of HEK-293 cells, stably expressing the *h*H_3_R. The results show that ST-2223 had high in vitro affinity for the desired targets, i.e., *h*H_3_Rs (*K*_i_ = 4.8 nM), *h*D_2_Rs (*K*_i_ = 19.8 nM), and *h*D_3_Rs (*K*_i_ = 2.0 nM), with a ratio of *h*D_2_Rs/*h*D_3_Rs of 10 (Figure 1). Moreover, the observed in vitro results showed that ST-2223 displayed low affinity for *h*H_1_Rs (85.2 nM), *h*D_1_Rs (564 nM), *h*D_5_Rs (5064 nM), and neglectable inhibition of acetylcholine esterase enzyme (*ee*AChE, <60% at a concentration of 1000 nM of ST-2223, preliminary data) as well as butyrylcholine esterase enzyme (*eq*BuChE, <30% at a concentration of 1000 nM of ST-2223, preliminary data).The in vitro results for the selectivity profile of ST-2223 demonstrate that *h*H_1_Rs, *h*D_1_Rs, hD_5_Rs, and AChE as well as BuChE enzymes are not involved in the observed in vivo behavioral enhancements. 

### 3.2. In Vivo Ameliorative Effects of ST-2223 on Behaviors of Treated BTBR Mice 

BTBR mice display a typical behavioral profile, characterized by robust social and communication deficits, together with increased RRBs [53]. Collectively, these traits have promoted the widespread use of this model in preclinical research to mimic the symptoms and generate novel hypotheses about the origin and components of neurodevelopmental disorders such as ASD, and to envisage prospective pharmacological modalities for the future therapeutic management of ASD. Therefore, the objective of this study was to assess the capability of the multi-targeting ligand ST-2223, incorporating H_3_R and D_2_R/D_3_R antagonist properties, to ameliorate RRBs and the alternation behaviors observed in BTBR mice. Following numerous investigations of neurochemical pathways in the brain to better understand the pathophysiology of ASD, the dysfunction of several neurotransmitter systems has been shown to be implicated in ASD [54]. This evidence suggests that alterations in neurotransmission plays a crucial role in ASD, and could be useful for prospective pharmacological intervention in this disorder. As alterations in histaminergic and dopaminergic [53] neurotransmission are thought to be involved in the phenotypic outcomes of ASD-related behavioral features [25,28,39,53,55], the present experiments investigated whether the pharmacological modulation of brain HA and DA—using the novel multiple-targeting H_3_R and D_2_R/D_3_R antagonist ST-2223—is capable of reducing RRBs in the BTBR mouse model of ASD. These conducted experiments allowed us to test the stereotyped repetitive behavior and behavioral rigidity through cognitively demanding insistence on sameness behaviors. Similarly to comparing the repetitive and cognitive inflexibility observed in ASD individuals vs. non-ASD individuals, BTBR mice displayed increased RRBs compared to B6 mice; B6 mice were used in the current study as normosocial comparators. Recent studies have suggested that histaminergic signaling abnormalities may contribute to a rare neurodevelopmental Tourette syndrome, characterized by stereotypies that are reported to be among the most prevalent comorbidities associated with ASD [29,32,56]. Moreover, a previous study revealed that activation of H_3_Rs is implicated in triggering stereotypies in a mouse model of tic disorder with repetitive behavior-related pathologies [57]. Relative to BTBR mice, B6 mice displayed low levels of marble burying, and this observation was consistent with several previous preclinical studies (Figure 2) [47,58]. 

In the current study, acute systemic treatment of BTBR mice with ST-2223 (2.5, 5 or 10 mg/kg, i.p.) or with the reference drugs MEM (5 mg/kg, i.p.) or ARP (1 mg/kg, i.p), resulted in comparable reductions in stereotyped repetitive behavior in the MBT test. The latter finding suggests that the functioning of auto and/or hetero H_3_Rs in BTBR attenuated certain RRBs. Notably, patients diagnosed with ASD have been found to exhibit significant impairment in executive functioning, with some authors suggesting that deficits in cognitive flexibility and set-shifting are consistently associated with the presence of RRBs in ASD [59]. Additionally, mounting evidence indicates that, along with HA release, the functioning of H_3_Rs as hetero-receptors can also regulate the release of several other brain neurotransmitters, including DA, ACh, Glu, 5-HT, and GABA, in several brain regions [60,61]. Thus, central H_3_R antagonism has been proposed to improve the cortical fast rhythms closely associated with cognitive behaviors [61]. Interestingly, a previous clinical study reported that neurocognitive deficit may selectively underlie the strong need for sameness in routine and in the environment that is exhibited by many individuals with ASD [8]. As a result, their findings related to the specific association of neurocognitive deficits to RRBs and the need for sameness, are supported by the results observed in the current study. Preclinically, numerous studies have indicated that H_3_R antagonists in particular exhibited a unique feature in the form of their potential cognition-enhancing property [19,20,50,62,63,64,65,66], suggesting their potential therapeutic role in the treatment of RRBs. In accordance with this, a previous report showed that mice with valproic acid (VPA)-induced ASD displayed reduced repetitive behavior in the MBT following treatment with the H_3_R antagonist ciproxifan [55]. Interestingly, the results observed for ST-2223 in the MBT are in agreement with a previous study, in which adult male Tuck-Ordinary mice with VPA-induced ASD features were sub chronically pretreated with the non-imidazole H_3_R antagonist DL77, yielding a significant and dose-dependent (5, 10, 15 mg/kg, i.p.) reduction in the percentage of marbles buried [67]. These previous observations are in harmony with the current results of MBT which comprehend the capability of ST-2223 to modulate the neurotransmission of HA and DA, supporting their crucial role in repetitive/compulsive behaviors in mice. Notably, most of the previous studies focused on effects of H_3_R antagonists on ASD, however, the current series of behavioral experiments are the first to assess the effects of a multiple-active H_3_R and D_2_R/D_3_R antagonist on behavioral parameters altered in ASD. Comparable to observations with MBT, ST-2223 (5 and 10 mg/kg) significantly reduced the self-grooming duration in BTBR mice, with no effect of the highest dose of ST-2223 on locomotor activity (Figure 3). Similar results were observed with reference drug ARP (1 mg/kg), suggesting that the mechanism by which the self-grooming effect is improved, following administration of ST-2223, may involve its potent antagonistic interactions with D_2_R/D_3_R, which may modulate the levels of DA in several specific brain areas. Moreover, ARP is considered as a dopamine stabilizer, as when DA levels are high, it acts as a D_2_R antagonist, and at low endogenous DA levels, it acts as a D_2_R agonist [68]. The involvement of DA in self-grooming, as suggested by the present data, is supported by a recent study which demonstrated the attenuation of enhanced frequency of self-grooming induced by anorexigenic peptide neuromedin U following systemic pretreatment with ARP, as neuromedin U was reported to increase the ex vivo level of DA [68]. As previously reported by Rapanelli et al. (2017), the pathology of repetitive behaviors in mice derives from a deficiency of brain HA. Markedly elevated grooming was observed by specific ablation or chemogenetic silencing of histaminergic neurons in the tuberomammillary nucleus (TMN) of the hypothalamus, and the detection of elevated neuronal activity markers was observed in both the dorsal striatum and the medial prefrontal cortex [69]. However, their findings showed that direct infusion of HA into the striatum reversed this behavioral pathology [69]. In the current study, the addressed H_3_R antagonistic effects of ST-2223 in alleviating self-grooming in BTBR mice are in line with those previously observed, namely its enhancement effect on the release of HA and other neurotransmitters in several brain regions. 

Consequently, assessing the levels of different brain neurotransmitters, including HA and DA, in different brain areas of BTBR mice with ASD-like behaviors, as well as after pre-treatment with ST-2223, would further help to understand which neural circuits may be involved in this observed improvement in self grooming. In addition, our current observations demonstrate that the NMDAR antagonist MEM significantly rescued self- grooming in BTBR mice. These results are in accordance with a previous study in which suppression of elevated NMDAR function in a VPA mouse model of ASD was found to normalize repetitive behaviors [70]. However, in B6 mice, there was no appreciable trend for ST-2223 in altering the normal level of self-grooming, while MEM tended to decrease self-grooming without altering the locomotor activity of B6 mice, as observed in the open field test (Figure 3 and Figure 5). The latter observations may explain the specific involvement of NMDAR and gluatamate transmission in self-grooming in dysregulated and normal conditions. Notably, self-grooming is an innate behavior and is the most frequently occurring awake behavior displayed by laboratory mice [71,72]. When the grooming patterns is not in a ritualistic manner and occurs sequentially and systematically, it resembles compulsive-like behaviors expressed in some ASD individuals and in other psychiatric disorders [71,72]. ST-2223 (5 and 10 mg/kg) decreased the elevated level of self-grooming of the whole body that is significantly elevated in BTBR mice reflecting RRB. On the other hand, all doses of ST-2223 showed no effect on the normal self-grooming displayed by B6 control mice, excluding any confounding effect of the test compound. Moreover, ST-2223 was unable to restore hyperactivity in BTBR mice, excluding any drug-induced depression like behaviors, or impaired motor function. Our observations are in agreement with the reported principal symptoms that characterize clinical depression seen in stressed animals, including decreased motor activity [73].

Neurocognitive deficit is clinically reported to be one of the selective mechanisms underlying the strong need for sameness in routine that is exhibited by many individuals with ASD [8]. Therefore, a Y maze assessing the spontaneous alternation is commonly used to evaluate cognition and working memory, as mice must recall the most recently visited arm. In line with this idea, the findings from Y maze behavioral assessments showed that ST-2223 and MEM enhanced cognitive performances by reversing the reduced percentage of spontaneous alternation behavior in BTBR mice, as decreases in the percentage of alternation can be attributed to same arm repeated visits reflecting cognition and attentional deficits (Figure 4). The B6 control animals showed high spontaneous alternations, suggesting a high randomness of their behaviors. Griebel et al. (2012) reported that SAR110894, a potent histamine H_3_-receptor antagonist, reversed a deficit in working memory in the Y maze test, following an acute low dose of phencyclidine (PCP) in mice sensitized by repeated treatment with a high dose of PCP [74]. Taken together, the relationship between working memory and cognitive abilities suggests that the H_3_R antagonist may be effective in treating RRBs in ASD, due to its therapeutic effects on certain aspects of cognition. Previous studies have reported that MEM rescued both social deficits and repetitive behaviors, such as self-grooming and jumping, in a VPA mouse model of ASD [70]. In agreement that suppression of elevated NMDAR functioning normalizes repetitive behaviors, here, we found that MEM significantly attenuated the reduced spontaneous alternation in BTBR mice, suggesting the observed reduction in sameness of the animal behavior. Accordingly, these collective observations reveal that the histaminergic and dopaminergic systems cross react or overlap in targeting the mechanistic RRBs of ASD; similarly effective as modulation of NMDA receptor functioning in BTBR mice.

The effects of ST-2223 on locomotor activity, as well as anxiety levels, were examined in order to exclude any false-positive effect in the behavioral tests. The results observed indicated that systemic treatment with ST-2223 in control B6 mice has no aversive effects in the open field test, indicating that ST-2223 does not change the baseline levels of motor, exploratory, or anxiety behaviors. However, ST-2223 significantly increased the amount of time spent by BTBR mice in the central arena, confirming the ability of ST-2223 to modulate anxiety-associated fear levels. In contrast, ST-2223 failed to restore hyperactivity, as no effect was exhibited with all doses on the total distance travelled (Figure 5). Consequently, and because ST-2223 treatment had no effect on locomotor activity in BTBR mice, the drug-induced reduction in marble burying and alternation behaviors cannot be explained by a more general reduction in activity. Instead, the results suggest that regulation of histaminergic and dopaminergic systems selectively modulated RRBs in BTBR mice. In contrast with a previous study [75], MEM failed to show significant anxiolytic effects in the open field test, a discrepancy that may be explained with the difference in dose regimen or anxiety level state in different mouse strains. Moreover, the failure of ST-2223 (at all doses), MEM and ARP to restore the hyperactivity observed in BTBR mice may have been due to the excitatory (Glu) and inhibitory (GABA) neurotransmitter imbalance which is well known to exist, as such an imbalance was observed in several clinical trials in patients with ASD [76,77,78,79]. However, a previous randomized clinical trial showed that following 6 weeks of treatment of MEM, this may be considered as an alternative to methylphenidate in children with attention deficit hyperactivity disorder (ADHD), characterized by lack of attention and hyperactivity [80]. Taken together, the current collective observations suggest that chronic treatment with MEM may be effective with regard to reducing anxiety and hyperactivity.

The strong ASD-like behavioral profile of BTBR mice, i.e., abnormalities in neurotransmitter systems in addition to several physiological and neurological features resembles that observed in patients diagnosed with ASD [81]. The latter observation suggests that the promising results of our current study on RRBs in BTBR mice may be a new therapeutic approach in the treatment of the core symptoms in ASD patients.

### 3.3. In Vivo Mitigating Effects of ST-2223 on Oxidative Stress in Different Brain Areas of Treated BTBR Mice

Another major objective of the current study was to test the capability of the most promising dose of ST-2223 (5 mg/kg) to mitigate abnormal levels of oxidative stress in four different brain areas of treated BTBR mice, namely the cerebellum, hippocampus, prefrontal cortex, and striatum. In the current study, the results showed that BTBR mice with ASD-like behaviors displayed significant increases in MDA levels, connected with a decline in GSH, SOD, and CAT levels in different brain parts of tested BTBR mice and as compared to control mice B6. Moreover, systemic administration with the most promising dose of ST-2223 (5 mg/kg), ARP (1 mg/kg) or MEM (5 mg/kg) showed a significant reduction in MDA, as well as a significant elevation in GSH, SOD, and CAT in different areas of the brain. The results observed on the levels of oxidative stress markers are in agreement with several previous reports from our group and other research groups that investigated the effects of H_3_R antagonists on oxidative stress in the brains of different rodent models [79,82,83,84,85,86]. Accordingly, previous reports showed that imidazole-based H_3_R antagonists, including clobenpropit and ciproxifan, mitigated several oxidative stress markers (e.g., MDA and GSH) in amphetamine- or dizocilpine-augmented oxidative stress in a preclinical mouse model of schizophrenia, suggesting the protective effect of H_3_R antagonists in such disease conditions [87,88,89]. Furthermore, systemic co-administration with RAM (10 mg) counteracted the ST-2223 (5 mg)-induced modulating effects on MDA, GSH, SOD, and CAT in BTBR mice, indicating that modulation of brain HA—provided by ST-2223—may have contributed to the correction of an unbalanced ratio of radical oxygen species through the generation of endogenous cellular antioxidant defensive mechanisms.

## 4. Materials and Methods

### 4.1. Animals

Male C57BL/6J (B6) and BTBR T+ tf/J (BTBR) mice (aged 8–10 weeks, weighing 25–35 g) (Jackson Laboratory, Bar Harbor, USA) from the central animal facility of the College of Medicine and Health Sciences, United Arab Emirates University [90] were utilized for all the in vivo assessments. All mice were housed in plastic cages in a temperature-controlled room (22–25 °C) on a standard 12 h light/dark cycle (lights on at 6 a.m.). The animals had free access to tap water and a standard rodent chow diet in their home cages. Experiments were performed during the light cycle. All experimental procedures described herein were performed according to the recommendations of the European Communities Council Directive of 24 November 1986 (86/609/EEC). All experiments were approved by the Institutional Animal Ethics Committee in the College of Medicine and Health Sciences/United Arab Emirates (Approval No. ERA-2017-5603).

### 4.2. Drugs

The drugs tested included ST-2223 [*N*-(4-(4-(2-methoxyphenyl)piperazin-1-yl)butyl)-6-(3-(piperidin-1-yl)propoxy)-2-naphthamide] that was designed and synthesized in the Institute of Pharmaceutical and Medicinal Chemistry, Heinrich Heine University Düsseldorf, Germany, and in accordance with previously described methodologies [44,45,91] (Supplementary Materials). Aripiprazole (ARP) (1 mg/kg, i.p.) and memantine (MEM, 5 mg/kg, i.p.), as reference drugs, were purchased from Sigma-Aldrich (St. Louis, MO, USA). The CNS-penetrant H_3_R agonist (*R*)-α-methylhistamine (RAM, 10 mg/kg, i.p.) was purchased from Sigma-Aldrich, St. Louis, MO, USA. T. Protease and the phosphatase inhibitor cocktail were procured from Thermo Scientific, USA. The assay kit for reduced glutathione (GSH) was obtained from Sigma-Aldrich (St. Louis, MO, USA). The lipid peroxidation assay kit for estimation of malondialdehyde (MDA) was purchased from North West Life Science (Vancouver, WA, USA). The assay kits for superoxide dismutase (SOD) and catalase (CAT) were purchased from Cayman Chemical (Ann Arbor, MI, USA). The drugs were prepared daily using 1% dimethyl sulfoxide (DMSO) in 0.9% saline, for intraperitoneal (i.p.) administration at a volume of 10 mL/kg of body weight. Vehicle treatment consisted of 1% DMSO in 0.9% normal saline. All drugs were i.p. administered 30 min before the behavioral test. The doses of these drugs were chosen based on previous reports. Neurobehavioral assessment was performed blindly with respect to experimental group and drug administration.

### 4.3. In Vitro Pharmacological Binding Assays for ST-2223 

In accordance with previous experimental protocols, the binding affinity of ST-2223 for H_3_Rs was assessed by applying the [^3^H]*N*^α^-methylhistamine binding assay, performed with cell membrane preparation of HEK cells stably expressing the human H_3_R (*n* = 4) [45,50,92,93,94]. In addition, and as previously described, the binding affinity of ST-2223 for human dopamine *h*D_1_Rs and *h*D_5_Rs (HEK) against [^3^H]SCH23390 and *h*D_2_SRs, *h*D_3_Rs (CHO) using [^3^H]spiperone (*n* = 3) was assessed (*n* = 3) [44,91]. Moreover, the inhibitory effects of the tested compound ST-2223 on acetylcholinesterase (AChE) and butyrylcholin esterase (BuChE) were determined using a modified Ellman’s method [46].

### 4.4. In Vivo Behavioral Tests

#### 4.4.1. Marble Burying Task (MBT)

The MBT is used for testing repetitive and compulsive behaviors as it accurately reflects repetitive digging behavior [95,96]. The test was performed as described previously, with slight modifications [96,97,98]. Briefly, cages (26 cm × 48 cm × 20 cm) were filled with fresh, unscented mouse bedding material to a depth of 5 cm, on top of which 20 marbles were placed in 4 rows of 5 marbles each (15 mm diameter). Before placing the marbles, the bedding surface was leveled by placing another cage of the same size onto the surface of the bedding and then each mouse was individually added for 10 min habituation. After habituation, the mouse was removed, and the marbles were carefully overlaid. The same mouse was then returned to its designated test cage and allowed to explore for 30 min. The total number of marbles buried (>50% marble covered by the bedding material) was determined at the end of the session [95].

#### 4.4.2. Self-Grooming Paradigm (SGP)

In the SGP test, mice were scored for spontaneous grooming behaviors. Self-grooming may represent more of a repetitive motor pattern that does not have a predominant cognitive component as with reversal learning [99]. To measure SGP, each mouse was placed individually in a standard clear plastic cage (28 cm wide × 17 cm long × 12 cm high) for a total of 20 min. Mice were allowed to freely explore the cage for the entire test, the first 10 min served as a habituation period. After 10 min habituation, each mouse was scored with a stopwatch for 10 min for cumulative time spent grooming all body regions. Grooming behavior included head washing, body grooming, genital/tail grooming and paw and leg licking. The observer sat approximately 1 m from the test cage during the second 10 min of testing [99].

#### 4.4.3. Spontaneous Alternation Behavior (SAB)

The SAB test is based on the exploratory strategy of rodents to explore a new environment. In examining repetitive behaviors, the Y maze test took advantage of the rodents’ natural tendency to spontaneously alternate or choose a different arm of the maze instead of the one they visited on their previous entry (i.e., least recently visited arm). A decrease in the percentage alternation can also be attributed to same arm repeated visits [100]. Spontaneous alternation behavior was measured on a Y maze apparatus, as previously described, with slight modifications. The Y maze composed of three arms (30 cm × 6 cm × 15 cm), where the arms were labeled with letters: A, B, or C. The test mouse was placed in the center of the maze and was allowed to freely explore the maze for 8 min. The number of entries into each arm and the total number of entries were recorded by the observer. A spontaneous alternation was defined as successive entry into three arms on an overlapping triplet set. Percentage spontaneous alternation was calculated as the number of spontaneous alternations (actual alternations) over the total number of entries (possible alternations). Two arm entries were subtracted from the denominator because an alternation required at least 3 entries. The apparatus was cleaned with 70% alcohol solution after each mouse was tested [101].

(1) $$\mathrm{Percentage}\;\mathrm{spontaneous}\;\mathrm{alternations}=\frac{\mathrm{Total}\;\mathrm{alternations}}{\left(\mathrm{Total}\;\mathrm{arm}\;\mathrm{entries}\;-\;2\right)}\;\times 100$$

For example, the sequence C, B, A, B, C, B, A, C, B (starting in arm A) resulted in a percent spontaneous alternation of 5/7 = 71.4%. 

#### 4.4.4. Locomotor Activity 

The open field test (OFT) systematically evaluates exploration behaviors executed in a novel open field (considered as a novel environment), as an independent control for the effects of the drugs on physical activity that could confound the interpretation of the results from the self-grooming, anxiety and spontaneous alternation. Mice were allowed to freely explore an open-field arena (45 × 45 × 30 cm), to assess general locomotor activity, and anxiety-related behaviors [82]. The center arena was defined as the central 23 × 23 cm area. Mice were given 5 min to habituate in the center area of the arena before the recording started for the actual behavioral assessments. Recording lasted for 10 min using charge-coupled device (CCD) camera-assisted motion tracking apparatus and software (EthoVision 3.1, Noldus Information Technology, The Netherlands), and the total distance moved in the whole arena, time spent in the center and periphery were recorded. Test chambers were cleaned with 70% ethanol after each trial and were allowed to dry until ethanol evaporation and odor dissipation had occurred. When interpreting the results observed in this assessment, less time spent in the center was considered as an indicator for high levels of anxiety-like behaviors and total distance travelled signified the overall locomotor activity of tested animals [98,102,103].

### 4.5. Brain Collection and Tissue Processing for Biochemical Analyses

At the end of the behavioral assessments, the animals were sacrificed following previously published protocols [67,84]. Animals were anesthetized with pentobarbital (40 mg/kg, body weight, i.p.), sacrificed, and perfused via intracardial infusion to wash out the blood, using 1× PBS (0.01 M phosphate buffer, 0.0027 M potassium chloride and 0.137 M sodium chloride) at pH 7.4. The blood removal was confirmed by the observation of whitish color liver, heart, and kidney, indicating that they were blood free. The brains were then quickly removed and placed on an ice plate. The cerebellum, hippocampus, prefrontal and striatum were excised from the brain and snap-frozen in liquid nitrogen for further use in biochemical assessments [67,84]. On the day of the biochemical tests, the tissues were homogenized using radioimmunoprecipitation assay (RIPA) buffer (50 mM Tris HCl, pH 7.4, 140 mM NaCl, 1 mM EDTA, 0.5% Triton X-100 and 0.5% sodium deoxycholate) supplemented with protease and phosphatase inhibitors. Then, the homogenates were centrifuged at 14,000 rpm for 30 min (4 °C), and the supernatant was used for evaluation of lipid peroxidation, glutathione, and antioxidant enzymes using spectrophotometric measurements and enzyme-linked immunosorbent assays (ELISA). [90,104].

#### 4.5.1. Oxidative Stress Marker Estimations

##### Malondialdehyde (MDA) Assay

This assay was performed to estimate the amount of lipid peroxidation in experimental animals using an MDA detection kit following the manufacturer’s instructions, as previously described in our laboratories [84,105,106]. Briefly, 250 μL of sample or calibrator was incubated with thiobarbituric acid, followed by rigorous vortexing. After 1 h incubation at 60 °C, the mixture was centrifuged at 10,000× *g* for 2–3 min and the reaction mixture was transferred to a cuvette. Spectra were measured at 532 nm and the results are expressed as µM MDA/mg protein.

##### Quantification of Glutathione (GSH)

Levels of GSH in tissue homogenates were carried out according to the manufacturer’s instructions of the commercially available Sigma’s glutathione assay kit (Sigma-Aldrich Chemie GmbH, Steinheim), and as previously described [84,105,106]. In brief, samples were first deproteinized with 5% 5-sulfosalicylic acid solution, centrifuged to remove the precipitated protein, and then the supernatant was used to estimate GSH. Ten microliter samples or standards were incubated with 150 μL of working mixture (assay buffer + 5,5′-dithiobis (2-nitrobenzoic acid) + GSH reductase) in 96-well plates for 5 min. Diluted NADPH solution (50 µL) was added into each well and mixed thoroughly. Absorbance was measured at 412 nm with the kinetics for 5 min by using the microplate reader. Results are expressed as µM GSH/mg protein. 

#### 4.5.2. Assay of Antioxidant Enzymes Activities

To assess the activity of antioxidant enzymes Superoxide dismutase (SOD) and Catalase (CAT), commercially available kits were used, following the manufacturer’s instructions, and as previously reported [105,106]. Cayman assay kits (Cayman Chemicals Company, Ann Arbor, MI, USA) were used to assess antioxidant enzyme (superoxide dismutase (SOD) and catalase (CAT)) activities in experimental animals. Catalase assay: Twenty microliters of samples or standards and 30 μL of methanol was added to the assay buffer (100 μL) in 96-well plates. To this mixture, twenty microliter of hydrogen peroxide was added and incubated for 20 min at room temperature (RT) to initiate the reaction. Following incubation, 30 μL of potassium hydroxide was used to terminate the reaction, followed by subsequent addition of catalase purpald (30 μL) and catalase potassium periodate (10 μL). The plate was incubated for 5 min at room temperature in a shaker and the plate was read at 540 nm using a microplate reader. Catalase activity was expressed as nmol/min/mg protein. Superoxide dismutase assay: ten microliters of sample or standard was added in 96-well plates. Twenty microliters of xanthine oxidase were added to initiate the reaction. The reaction mixture was mixed for few seconds and incubated (covered) for 30 min at room temperature (RT). Absorbance was read at 450 nm using microplate reader. The activity of SOD was expressed as units/mg protein. 

### 4.6. Statistics

Separate two-way analyses of variance ANOVAs (strain: B6, BTBR; treatment: vehicle, 2.5, 5, 10 mg/kg ST-2223) were conducted for biochemical assessments, marble burying, self-grooming, alternation behaviors, and locomotor activity. A significant interaction was followed by Tukey HSD post hoc tests to determine significant treatment differences in both strains. For statistical comparisons, the software package SPSS 25.0 (IBM Middle East, Dubai, UAE) was used. *p* values less than 0.05 were considered statistically significant.

## 5. Conclusions

The novel multiple-active H_3_R and D_2_R/D_3_R antagonist ST-2223 ameliorated ASD-like RRBs that are naturally exhibited in the BTBR mouse model of ASD. To the best of our knowledge, this is the first time the effects of the interplay between HA and DA on ASD-like RRBs have been explored directly. The in vivo demonstration of the effectiveness of a potent multiple-active test compound in palliating the RRBs observed in BTBR mice, provides evidence for the potential role of such compounds in treating ASD. Moreover, ST-2223 modulated the increase in the level of MDA and the decrease in levels of GSH, SOD and CAT in the cerebellum, hippocampus, as well as the prefrontal cortex, providing sufficient evidence that oxidative stress balance plays a crucial role in the severity of ASD-like RRBs in BTBR mice. Given the fact that there are no FDA approved effective treatments for the core symptoms of ASD, identification of novel treatments is necessary. As our treatment consisted of a single dose, further studies are still necessary to determine whether chronic treatment or repeated administration of ST-2223 is more effective than the acute dose, and whether this could possibly even extend the duration of the positive effects of the acute dose. The consistent autism-relevant behavioral phenotype of BTBR is an invaluable tool in deciphering ASD’s complex pathophysiology and discovering valid treatment methods. Therefore, the results of our current study support the validity of the translational value for the possible clinical applicability of multiple-active H3R and D2R/D3R antagonists, e.g., ST-2223, in the modulation of RRBs in ASD and several other neuropsychiatric diseases.

## Figures and Tables

**Table 1:** ST-2223 mitigated levels of oxidative stress markers in different brain parts of treated BTBR mice.

		BTBR				
	B6 (VEH)	(VEH)	ST-2223 (5 mg/kg)	ST-2223 (5 mg/kg) + RAM (10 mg/kg)	ARP (1 mg/kg)	MEM (5 mg/kg)
**MDA**						
Cerebellum	29.9 ± 3.3	54.0 ± 6.4 ^##^	35.2 ± 1.8 **	45.0 ± 0.6 ^$$^	36.8 ± 1.8 ***	26.7 ± 3.6 ***
Hippocampus	35.1 ± 3.0	53.8 ± 2.9 ^##^	38.1 ± 3.1 **	40.7 ± 3.3	32.9 ± 4.1 ***	32.8 ± 3.4 ***
Prefrontal Cortex	41.1 ± 7.0	58.3 ± 1.8 ^#^	39.1 ± 3.2 ***	53.6 ± 1.2 ^$$^	43.0 ± 2.5 **	40.0 ± 5.7 **
Striatum	30.8 ± 4.1	52.7 ± 3.4 ^##^	34.5 ± 5.5 *	51.4 ± 4.8 ^$^	41.2 ± 1.4 *	32.6 ± 4.1 **
**GSH**						
Cerebellum	1560 ± 103.5	827 ± 93.3 ^##^	1108 ± 41.1 *	1266 ± 70.2	1030 ± 36.2 *	877 ± 55.7
Hippocampus	2119 ± 425.1	1096 ± 16.7 ^#^	916 ± 4.2	643.6 ± 49.9	611.5 ± 62.6 ***	642.6 ± 66.0 ***
Prefrontal Cortex	1776 ± 266.6	835 ± 97.4 ^#^	1212 ± 302.6 *	1070.4 ± 64.8 ^$^	935 ± 98.0	719.3 ± 50.0
Striatum	1128 ± 199.5	583 ± 68.8 ^#^	1007 ± 93.1 *	735.5 ± 59.9 ^$^	1139 ± 74.6 *	915.5 ± 97.3 *
**SOD**						
Cerebellum	60.6 ± 3.1	49.7 ± 1.3 ^#^	64.3 ± 1.0 ***	49.2 ± 2.0 ^$$$^	56.4 ± 1.1 **	64.4 ± 3.1 **
Hippocampus	66.4 ± 5.4	49.5 ± 0.5 ^#^	48.1 ± 4.2	39.5 ± 0.5 ^$$^	51.8 ± 2.0	41.4 ± 3.4
Prefrontal Cortex	53.8 ± 2.2	45.1 ± 2.8 ^#^	43.2 ± 3.4	42.9 ± 4.2	54.0 ± 5.2 **	44.9 ± 2.2
Striatum	68.5 ± 4.7	51.8 ± 1.2 ^#^	51.2 ± 7.7	45.0 ± 1.0	65.7 ± 7.7 **	66.47 ± 4.4 **
**CAT**						
Cerebellum	156.7 ± 7.29	130. 7 ± 3.3 ^#^	120.6 ± 8.8	153.3 ± 5.4	159.2 ± 5.1 **	132.3 ± 4.5
Hippocampus	131.7 ± 3.4	111.4 ± 4.8 ^#^	173.0 ± 1.9 *	100.6 ± 3.6 ^$$^	118.3 ± 6.5	114.3 ± 11.2
Prefrontal Cortex	130.3 ± 9.4	80.1 ± 14.9 ^#^	116.8 ± 5.3 *	70.9± 3.9 ^$^	96.2 ± 1.3	94.1 ± 4.8
Striatum	117.7 ± 2.5	104.6 ± 2.28 ^#^	119.1 ± 3.7 *	76.0 ± 9.3 ^$$^	120.5 ± 10.1 *	108.1 ± 3.4

Malondialdehyde (MDA), glutathione (GSH), catalase (CAT), and superoxide dismutase (SOD) were assessed in four different brain regions of treated BTBR mice, namely, the cerebellum, hippocampus, prefrontal cortex and striatum. BTBR mice showed a significant increase in MDA and significant decrease in GSH, SOD, and CAT compared to B6 mice. The effects of systemic administration of ST-2223 (5 mg/kg) were observed in B6 and BTBR mice. ST-2223 (5 mg/kg) significantly reduced the increased levels of MDA and significantly increased the reduced levels of GSH, SOD and CAT. Data are expressed as the mean ± SEM (*n* = 3–6). ^#^ *p* < 0.05 vs. B6 mice. ^##^ *p* < 0.01 vs. B6 mice. * *p* < 0.05 vs. BTBR mice. ** *p* < 0.01 vs. BTBR mice. *** *p* < 0.001 vs. BTBR mice. ^$^ *p* < 0.05 vs. ST-2223 (5 mg)-treated BTBR mice. ^$$^ *p* < 0.01 vs. ST-2223 (5 mg)-treated BTBR mice. ^$$$^ *p* < 0.001 vs. ST-2223 (5 mg)-treated BTBR mice. In biochemical assessments, 6 groups of 3–6 mice per group were used. The effects of ST-2223 were analyzed using two-way analysis of variance (ANOVA) with dose of drugs and animals (either BTBR or B6 mice) as the between-subjects factor, and post hoc comparisons were performed with Tukey’s test in case of a significant effect. VEH: vehicle, RAM: (*R*)-α-methylhistamine, ARP: aripiprazole, MEM: memantine.

## Supplementary Materials

The following are available online at https://www.mdpi.com/1422-0067/22/4/1947/s1, Figure S1: Synthesis and analytics of ST-2223., Figure S2: ^1^H-NMR spectral results of ST-2223 in DMSO-d6.
