# Supplementary material for: The Multi-Targeting Ligand ST-2223 with Histamine H3 Receptor and Dopamine D2/D3 Receptor Antagonist Properties Mitigates Autism-Like Repetitive Behaviors and Brain Oxidative Stress in Mice
Source: Int J Mol Sci. 2021 Feb 16;22(4):1947. doi: 10.3390/ijms22041947 (PMC7920280; doi:10.3390/ijms22041947)
Supplement: Supplementary file 1 [file ijms-22-01947-s001.pdf]

Supplementary Materials

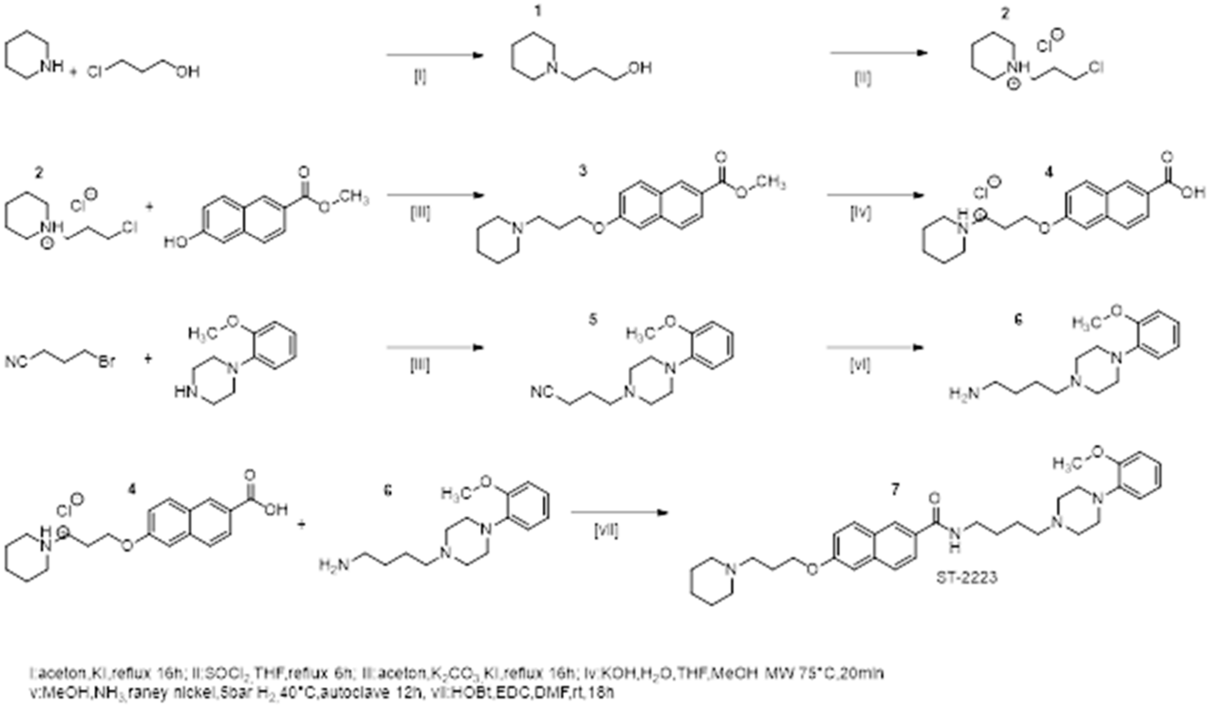

Figure S1. Synthesis and analytics of ST-2223.

All reagents and solvents were purchased by commercial suppliers. Mass spectra was determined with Advion CMS mass spectrometer using APCI. ^{1}H-NMR was measured by Bruker Avance III 300 MHz using DMSO-d6 as solvent. Results were displayed as chemical shifts δ in parts per million with solvents as internal standard. Multiplicity described as s: singlet, d: doublet, t: triplet, m: multiplet and number of protons. Melting points were determined with Büchi M-564. LC-MS was performed on Bruker Elute SP with Bruker amazon speed ESI mass spectrometer using an Intensity Solo C18 RP 100x2.1mm column. As solvents acetonitrile and water with 0,1% formic acid in LCMS quality were used. Results given as retention time t_R in minutes, area fraction in % and mass to charge ratio m/z. Substances were accepted as pure with more than 95% area fraction. Colum chromatography (CC) was performed with silica gel 60 (0.04-0.063) from Machery&Nagel with solvents as mentioned in technical quality. Flash chromatography was done with Biotage Isolera Spektra system with ACI and Assist. Biotage Sfär Silica D and Biotage Sfär Amino D were used as columns and solvents as mentioned in technical quality.

3-(Piperidin-1-yl)propan-1-ol 1[1]

Compound **1** was synthetized from piperidine (5.0ml; 50mmol). 3-chlorpropan-1-ol (4.2544g; 45mmol), potassium carbonate (13.8g; 100mmol) and potassium iodide (catalytic) in 150 ml acetone under reflux condition. Product was distilled at 20 mbar and 95°C. The oil was dissolved in isopropanol and precipitated with HCl in dioxane. Yield: 68%

APCI-MS: (+): m/z: 144.3/145.3^{1}H NMR (300 MHz, DMSO) δ 10.84 (s, 1H), 3.74 (t, *J* = 6.4 Hz, 2H), 3.38 (d, *J* = 12.1 Hz, 2H), 3.07 (dt, *J* = 12.3, 4.3 Hz, 2H), 2.93 – 2.75 (m, 2H), 2.29 – 2.15 (m, 2H), 1.93 – 1.62 (m, 5H), 1.37 (dtt, *J* = 17.5, 12.6, 6.7 Hz, 1H).

1-(3-Chloropropyl)piperidine hydrochloride 2[1]

Compound 1 was dissolved in tetrahydrofuran, thionyl chloride was added and stirred 5 h under reflux conditions. The reaction mixture was cooled to room temperature and diluted with ether. The participate was filtered off and washed with ether. Yield: 98.7%

APCI-MS: (+): m/z: 161.1/163.1 (M+H)+ 1H NMR (300 MHz, DMSO-d6) δ 10.88 (s, 1H), 3.74 (t, J = 6.4 Hz, 2H), 3.43 – 3.35 (m, 2H), 3.12 – 3.01 (m, 2H), 2.84 (tdd, J = 12.3, 9.0, 3.6 Hz, 2H), 2.27 – 2.14 (m, 2H), 1.93 – 1.62 (m, 5H), 1.36 (qt, J = 12.8, 4.6 Hz, 1H).

Methyl 6-(3-(piperidin-1-yl)propoxy)-2-naphthoate [2] 3

Compound **3** was synthetized via Williamson ether synthesis using methyl 6-hydroxy-2-naphthoate (2.997g; 14.8mmol), 1-(3-chloropropyl)piperidine (2.660g; 13.45mmol), potassium carbonate (9.20g; 67mmol) and potassium iodide (cat.) in acetone under reflux conditions for 16h. The solvent was filtered and evaporated. The residue was dissolved in DCM and washed three times with 1 N NaOH solution. The organic layer was dried with $MgSO_4$ and evaporated. Yield:86.2%

APCI-MS: (+): m/z: 328.5/329.5 $(M+H)^{+1}$H NMR 8.50 (dd, *J* = 1.8, 0.8 Hz, 1H), 7.98 (dt, *J* = 8.7, 0.8 Hz, 1H), 7.86 (dd, *J* = 8.7, 1.8 Hz, 1H), 7.77 (d, *J* = 8.6 Hz, 1H), 7.24 – 7.13 (m, 2H), 3.80 (s, 3H), 2.32 (m, 6H), 1.85 (dt, *J* = 7.6, 6.5 Hz, 2H), 1.47 (p, *J* = 5.5 Hz, 4H), 1.36 (q, *J* = 6.0 Hz, 2H

6-(3-(Piperidin-1-yl)propoxy)-2-naphthoic acid x HCl [2] 4

Compound **4** (3.80g; 11.6mmol) and potassium hydroxide (2.8095g; 50mmol) were dissolved in tetrahydrofuran, methanol and water. The mixture was filled in a microwave vial and heated in a microwave to 70°C for 15 minutes. The organic solvents were evaporated and the water phase was neutralized with concentrated hydrochloric acid. At pH 2-3, the product precipitated as isoelectric structure. Dissolving in dioxane and adding HCl gave the protonated compound. Yield: 90.2%

APCI-MS:(+):m/z: 328.5/329.5 (-):m/z:326.5/327.5

^{1}H NMR (300 MHz, DMSO-d_6) δ 10.81 (s, 1H), 8.52 (d, *J* = 1.6 Hz, 1H), 8.03 (d, *J* = 9.0 Hz, 1H), 7.97 – 7.84 (m, 2H), 7.43 (d, *J* = 2.5 Hz, 1H), 7.24 (dd, *J* = 9.0, 2.5 Hz, 1H), 4.22 (t, *J* = 6.1 Hz, 2H), 3.45 (d, *J* = 12.1 Hz, 2H), 3.26 – 3.13 (m, 2H), 2.87 (dh, *J* = 12.1, 3.7 Hz, 2H), 2.29 (dq, *J* = 11.6, 6.0 Hz, 2H), 1.94 – 1.66 (m, 5H), 1.39 (ddt, *J* = 12.6, 8.7, 4.2 Hz, 1H).

4-(4-(2-Methoxyphenyl)piperazin-1-yl)butanenitrile [3] 5

Compound **5** was synthetized via Williamson ether synthesis using 1-(2-methoxyphenyl)piperazine (1.75ml; 10mmol), 4-bromobutyronitrile (1.0ml; 10mmol), potassium carbonate (4.15g; 30mmol) and potassium iodide (cat.) in acetone under reflux conditions for 16h .The solvent was filtered and evaporated. The residue was dissolved in DCM and washed three times with 1 N NaOH solution. The organic layer was dried with $MgSO_4$ and evaporated. The product was purified using flash chromatography witch silica gel and dichloromethane: methanol (97:3-93:7) as eluent. Yield: 94,6%

APCI-MS: (+): m/z: 260.4/261,4 ^{1}H NMR (300 MHz, DMSO-d_6) δ 7.00 – 6.82 (m, 4H), 3.77 (s, 3H), 2.96 (s, 4H), 2.53 (d, *J* = 5.4 Hz, 2H), 2.51 (d, *J* = 1.9 Hz, 4H), 2.41 (t, *J* = 6.8 Hz, 2H), 1.76 (p, *J* = 7.0 Hz, 2H).

4-(4-(2-Methoxyphenyl)piperazin-1-yl)butan-1-amine [3] 6

Compound **5** (: 4.8321g; 8.6 mmol) was dissolved in methanol and some ammonia. Activated raney nickel was added and the mixture was stirred in an autoclave under 5bar hydrogen and 40°C for 12h. The

mixture was filtered over Celite® and the solvent was evaporated.Yield:89.4%

APCI-MS:(+): m/z: 264.5/265.5 ^{1}H NMR (300 MHz, DMSO-d_6) δ 6.97 – 6.81 (m, 4H), 3.76 (s, 3H), 2.94 (t, *J* = 4.7 Hz, 4H), 2.53 (dd, *J* = 10.1, 4.3 Hz, 2H), 2.48 (s, 4H), 2.29 (t, *J* = 7.2 Hz, 2H), 1.54 – 1.40 (m, 2H), 1.35 (dq, *J* = 8.7, 6.8 Hz, 2H).

N-(4-(4-(2-Methoxyphenyl)piperazin-1-yl)butyl)-6-(3-(piperidin-1-yl)propoxy)-2-naphthamide 7 (ST-2223)

Compound **6** (2.1067g; 8mmol), acid **4** (2.8001g; 8mmol), HOBt (1.2241g; 8mmol) and EDC (1.40ml; 8mmol) were dissolved in DMF and stirred for 18h at room temperature. DMF was reduced and diluted with water and 1 N NaOH solution. The mixture was three times extracted with ethyl acetate. The combined organic layers were dried with $MgSO_4$ and evaporated. The products was recrystallized from acetone. Yield: 61.8%

APCI-MS: (+): m/z: 557.9/558.9 $(M+H)^+$

Melting point: 131.7°C-132.2°C

^{1}H NMR (300 MHz, $CDCl_3$) δ 8.21 (d, *J* = 1.7 Hz, 1H), 7.85 – 7.75 (m, 2H), 7.74 (d, *J* = 8.6 Hz, 1H), 7.24 – 7.13 (m, 2H), 7.01 (td, *J* = 7.6, 1.7 Hz, 1H), 6.95 – 6.78 (m, 4H), 4.16 (t, *J* = 6.4 Hz, 2H), 3.86 (s, 3H), 3.55 (q, *J* = 6.1 Hz, 2H), 3.06 (s, 4H), 2.66 (d, *J* = 5.5 Hz, 4H), 2.59 – 2.39 (m, 8H), 2.07 (dq, *J* = 8.9, 6.5 Hz, 2H), 1.98 (s, 1H), 1.81 – 1.56 (m, 7H), 1.48 (q, *J* = 5.9 Hz, 2H).

^{13}C NMR (75 MHz, $CDCl_3$) δ 168.03, 158.49, 152.36, 141.33, 136.31, 130.44, 130.09, 128.06, 127.24, 127.19, 124.41, 123.04, 121.07, 120.05, 118.30, 111.26, 106.57, 77.58, 77.36, 77.16, 76.74, 66.73, 58.21, 56.12, 55.46, 54.79, 53.60, 50.64, 40.19, 27.63, 26.85, 26.05, 24.67, 24.52.

LC-MS: t_R: 14.0 min Area Frak.: 100.0% m/z: 280.19 $(M+2H)^{2+}$

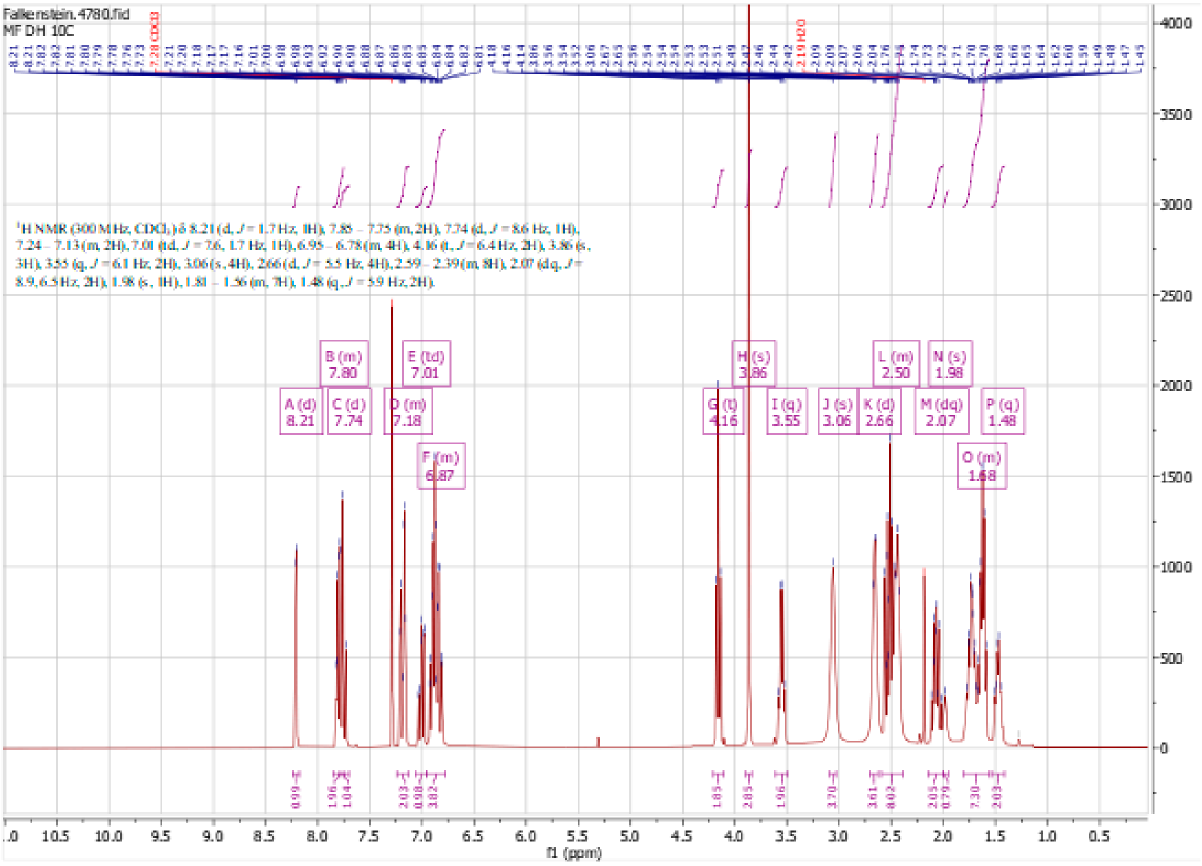

Figure S2. ^{1}H-NMR spectral results of ST-2223 in DMSO-d_6.

References:

1. Sander, K.; Kottke, T.; Weizel, L.; Stark, H. Kojic Acid Derivatives as Histamine H3 Receptor Ligands. *Chem. Pharm. Bull.* **2010**, *58*, 1353–1361, doi:10.1248/cpb.58.1353.
2. Gatti McArthur, S., Hertel, C., Plancher, J.-M., Roche, O. Naphtaline derivatives useful as histamine 3 receptor ligands. *Int. Appl. Publ. Under Pat. Coop.* **2005**, Treaty WO2005/117.
3. Hackling, † A.; Ghosh, ‡ R.; Perachon, § S.; Mann, ∥ A.; Höltje, ‡ H.-D.; Wermuth, ∥ C.G.; Schwartz⊥.J.-C.; Sippl, ‡ W.; Sokoloff⊥.A.P.; Stark, † H. N-(ω-(4-(2-Methoxyphenyl)piperazin-1-yl)alkyl)carboxamides as Dopamine D2and D3Receptor Ligands. *J. Med. Chem.* **2003**, *46*, 3883–3899, doi:10.1021/jm030836n.
